# Emoji or real emotions? The effect of digitalization on the quality of life through social media addiction – a multigroup SEM analysis, moderated by positive and negative affect

**DOI:** 10.1186/s40359-025-03508-z

**Published:** 2025-10-30

**Authors:** Daniela-Elena Lițan

**Affiliations:** Department of Psychology, West University of Timișoara, Timișoara, Romania

**Keywords:** Social media addiction, Quality of life, Positive affect, Negative affect, Multigroup SEM

## Abstract

**Background:**

This study examines how positive and negative affect moderate the relationship between social media addiction and quality of life, focusing on gender differences. It brings novelty by addressing these mechanisms in a culturally underrepresented context, Romanian adults, and integrates emotional regulation into the assessment of digital well-being.

**Methods:**

Data were collected via an online questionnaire from a sample of Romanian adults. Quality of life was modeled as a latent construct encompassing five dimensions: material and physical well-being, relationships with others, social and civic activities, personal development, and recreation. Multigroup Structural Equation Modeling (SEM) was employed to examine moderation effects across men and women.

**Results:**

The findings show that negative affect amplifies the detrimental impact of social media addiction on quality of life, while positive affect has a protective role. These effects are present in both genders but differ in intensity, with men showing stronger vulnerability to negative affect and women benefiting less from positive affect.

**Conclusion:**

The study highlights the importance of emotional regulation and gender-sensitive approaches when addressing social media addiction. The findings provide evidence-based directions for developing personalized interventions aimed at enhancing positive affect and improving emotional coping strategies to mitigate the negative consequences of excessive social media use.

## Introduction

Social networks are a modern concept through the ”lens” of technology, yet older than we might think. If we were to scroll through the pages of history, we would find the concept of Agora, which appeared in the 11th-9th centuries BC, specific to Ancient Greece - the political, social, economic and cultural center of Greek cities - the place where citizens met for political debates, trade, social events, public speeches, trials and fairs [1]. Appearing later, in the 8th century BC, the Roman Forum was in its turn the place where for more than 1000 years (until the fall of the Western Roman Empire (476 AD) [2]), the citizens learned about laws, political decisions and events, politicians, orators and community leaders gave speeches, news circulated through the voice of the people or was displayed on the Acta Diurna [3]. The Forum was also a place of entertainment, where parades, public trials, executions, fairs were held [4], and people could cheer, expressing their approval or dissatisfaction.

And here we are today! The Agora and the Roman Forum, a few thousand years later, have transformed, symbolically speaking, into the so-called social networks. Social networks can be defined as a set of interactive applications, connected to the Internet, that facilitate the creation, management and sharing of user-generated content [5]. With the development of digital technology, social networks have become “today”, the Forum of “yesterday”, with global coverage, due to the fact that people use them to inform themselves (news), to keep in touch with family and friends but also to make new friends, to promote businesses, to express their opinions as well as to hold debates on various topics, etc [6–8]., sometimes even influencing government or corporate decisions. In other words, social networks are a public space for communication, debate and influence. Given their pervasive role in modern life, understanding the psychological and social implications of social networks has become increasingly important. At the same time, concerns about maladaptive use and its broader consequences motivate a focused empirical inquiry.

With a total of 5.24 billion users or 63.9% of the world’s population in February 2025 [9], social media are used by people of all ages [10]. Romania, a Southeastern European country with a population of 19.1 million [11], follows the global trend: 17.3 million people are active on social media [12]. This accelerated digitalization, combined with the rapid adoption of mobile technologies, has fundamentally reshaped how individuals interact and manage their psychological well-being. While social media platforms provide unprecedented opportunities for information exchange and connectivity, their intensive use raises important concerns regarding potential negative consequences on mental health and quality of life. These issues are particularly relevant for Romania, where the vast majority of internet users are active on social media, making this study both timely and necessary. In this context, the construct of social media addiction was operationalized using the Social Media Addiction Scale [13], which assesses problematic patterns of smartphone and social media use through indicators such as salience (preoccupation with online activities), conflict/functional impairment (interpersonal or daily life difficulties due to excessive use), withdrawal-like symptoms (e.g., agitation when unable to access the device), and tolerance (spending more time than intended). While these indicators partially overlap with the components described in Griffiths’ behavioral addiction model [14, 15], they are adapted to the context of smartphone-based social media use. These components reflect not only problematic patterns of use but also highlight how social media can become a tool for compulsive emotion regulation, where individuals attempt to manage positive or negative affect through constant online engagement. In February 2024, the top social media preferences of Romanians were Facebook (46%), followed by YouTube (28%), WhatsApp (23%), TikTok (16%), Facebook Messenger (12%) and Instagram (11%) [16]. These penetration and usage patterns make Romania a pertinent setting for examining how intensive platform engagement relates to well-being.

From the desire to stay in touch with friends, to spend leisure time in a pleasant way, to be up to date with current news to finding communities with similar ideas [7], in the context of digitalization, the intensive use of social media can escalate into addiction, affecting the quality of life. From the specialized literature, we can infer that women and men may have different reasons for using social media. For example, women are often more likely to use social media for social interactions and emotional support [17], while men tend to use them more to find a partner [18, 19] and consume content [20, 21]. Thus, it is essential to analyze whether the relationship between social media addiction and the quality of life is influenced by gender. In this study, gender refers to participants’ self-identified categories (“men” and “women”), as reported in the demographic section, and is used as a proxy for potential socialized differences in motivations and emotional regulation. We do not investigate biological sex directly. We also consider the moderating roles of positive affect and negative affect in this association.

This study contributes to the literature in three key ways: (1) it provides empirical evidence from Romania, a country underrepresented in studies of social media addiction and well-being; (2) it integrates positive and negative affect as moderators, adding a deeper understanding of how emotional states influence the impact of social media addiction on quality of life; and (3) it explores potential gender differences, addressing the need for culturally sensitive insights into these mechanisms. Together, these elements position the study to address a regionally underrepresented context with a theory-driven, moderation-focused design.

Based on these considerations, the present study aims to examine how social media addiction relates to quality of life among Romanian adults, focusing on the moderating roles of positive and negative affect and potential gender differences.

## Literature review

This section outlines the theories underpinning the study and shows how they inform our objectives and hypotheses.

### Theoretical background

- Uses and gratifications theory (UGT)

In order to understand the mechanisms underlying social media addiction and its impact on the quality of life, the current research is based on the UGT. According to this theory [22], users select the media they consume according to the goals they want to achieve, integrating and assimilating media messages into their daily lives to obtain maximum satisfaction [23]. This means that users assess content according to their needs and gratifications [24].The UGT has been used for many years to understand the motivations for using traditional media, such as radio, television, newspapers and magazines, and over time it has also been applied to modern ways of communication [25, 26]. According to this theory, we understand that users choose digital media to satisfy certain psychological needs, such as [27–29]: social connection, information, personal development, stress avoidance and entertainment, affective needs (emotional gratifications). Beyond UGT, other theoretical frameworks conceptualize the psychological needs underlying social media use. For example, Self-Determination Theory (SDT) [30] emphasizes three basic needs, autonomy, competence, and relatedness, which can be partially fulfilled through online interactions. Similarly, the Need for Cognition framework [31] supports the idea that seeking information is itself a legitimate psychological need, especially in digital environments where knowledge acquisition and opinion formation are mediated by social platforms. Integrating these perspectives strengthens the theoretical grounding of this study, showing that needs such as information, social connection, and personal development have a well-established basis in the literature on motivation and well-being. Although social media offer these gratifications, their excessive use can lead to addiction, affecting the balance between digital and real life. Within this “lens”, intensive, need-driven engagement can become habitual or compulsive (social media addiction), displacing offline activities central to quality of life.

b) Affect mechanisms and gender rationale

In addition to UGT, we draw on two complementary affect-based frameworks: Broaden-and-Build (for positive affect) [32, 33] and Conservation of Resources (for negative affect) [34]. These theories do not belong to UGT; rather, they extend it by specifying when and for whom the social media addiction–quality of life association should be stronger or weaker.

A less explored aspect is whether the relationship between social media addiction and quality of life differs by gender.

Another key aspect of this study is the role of positive and negative affect in the relationship between social media addiction and the quality of life. negative affect (e.g., anxiety, stress, sadness) can intensify the negative effects of social media addiction, which can even affect sleep quality [35] and memory loss [36], while positive affect can moderate the negative effects of addiction, helping the individual maintain a balance between social media use and real life [37–39]. Furthermore, these effects may differ between women and men, given the differences in how emotions are processed and regulated in each category. According to the specialized literature, the differences between women and men in emotion processing have both biological bases: neuroscientific [40, 41] and hormonal [42, 43], as well as social bases influenced by education and cultural norms [44, 45]. Although these biological influences are acknowledged, our analyses focus on gender as reported by participants, which reflects socialized roles and expectations rather than biological sex per se. Therefore, these differences may influence how positive and negative affect moderate the relationship between social media addiction and the quality of life. Including both positive and negative affect as moderators provides a more nuanced understanding of when and for whom social media addiction is most detrimental.

These affective and gender-related mechanisms directly inform the moderation hypotheses presented in Hypotheses development. Documented gender differences in motives for social media use and in emotion processing/regulation further justify a multigroup test of these pathways [17–21, 46–52].

Taken together, these theoretical perspectives explain how intensive social media use may influence quality of life differently across individuals. By integrating emotional mechanisms (positive and negative affect) and gender-based differences into the model, the present study builds a conceptual framework that clarifies when and for whom social media addiction is most detrimental.

Prior research shows that intensive and problematic social media use can affect multiple dimensions of quality of life through different mechanisms. For example, excessive engagement with social platforms can reduce material and physical well-being by displacing time allocated to work, rest, and healthy routines [53, 54]. Similarly, constant online activity may undermine interpersonal relationships by reducing opportunities for face-to-face interactions and generating conflicts within close ties [55, 56]. Participation in social, community, and civic activities may also decline as online engagement replaces offline involvement [57]. Furthermore, problematic use can interfere with personal development and fulfillment, as it limits focus on self-improvement goals and academic or professional achievements [58]. Finally, excessive time spent on social media may reduce recreation quality, as activities aimed at relaxation are replaced with compulsive browsing [56, 58].

Accordingly, we anticipate: (i) lower material and physical well-being via sleep/work disruption and routine displacement; (ii) poorer relationships due to reduced face-to-face time and heightened interpersonal conflict; (iii) diminished social, community, and civic participation through online–offline displacement; (iv) reduced personal development and fulfillment via attentional capture and goal interference; and (v) lower recreation quality because compulsive, non-restorative browsing replaces restorative leisure [53–58].

### Hypotheses development

This subsection synthesizes the literature gaps to derive testable moderation hypotheses.

Although social media addiction has been extensively investigated in Western contexts, relatively few studies have explored its implications within Central and Eastern Europe. Despite the growing body of research on social media addiction, several critical gaps remain unaddressed. Beyond the geographical context, a closer examination of the existing literature reveals several empirical gaps. First, while previous studies have established associations between social media addiction, well-being, and quality of life, few have examined the underlying mechanisms explaining these relationships (e.g [59]).,. Second, the moderating roles of positive and negative affect remain largely unexplored, despite their theoretical importance for understanding individual differences in how people respond to intensive social media use (e.g [60]).,. Third, limited attention has been paid to gender-based differences in these dynamics, even though prior research suggests that motivations for social media use often diverge between women and men (e.g [61]).,. These research gaps are closely related to broader societal concerns about how intensive social media use influences psychological well-being and quality of life.

By addressing these underexplored aspects, the present study advances the literature beyond simple associations and offers a more nuanced, culturally informed understanding of these relationships. Exploring these underexamined mechanisms is important for building a comprehensive understanding of how social media addiction affects quality of life across diverse populations. Previous studies have contributed valuable insights into social media addiction and well-being, yet significant gaps remain, particularly regarding mechanisms, emotional moderators, and gender differences. By addressing this gap, our study provides insights that are both locally relevant and internationally significant. Although the study focuses on Romanian adults, the mechanisms explored are grounded in established theoretical frameworks, which increases the potential generalizability of our findings to other cultural and social contexts. At the same time, cultural and demographic differences should be considered when adapting these insights to different populations. Consistent with prior research, social media addiction has been linked to lower well-being and life satisfaction [62–64], which supports our hypothesis regarding its negative effects on quality of life.

Grounded in the theoretical frameworks detailed in Theoretical background, we expect that positive affect can buffer the negative impact of social media addiction on quality of life, whereas negative affect may exacerbate it. These mechanisms justify the moderation hypotheses tested in this study. Here, “moderation” means that the social media addiction → quality of life slope changes as a function of affect levels. Empirically, higher positive affect is linked to better health and functioning and reduced stressor impact [32, 33], whereas negative affect is associated with rumination and poorer psychological outcomes [13, 65–68]. In line with the gender rationale outlined in Affect mechanisms and gender rationale, we examine these relations separately for women and men using a multigroup SEM approach. This motivates two hypotheses:

- H1: Positive affect moderates the relationship between social media addiction and quality of life, such that higher positive affect diminishes the negative impact for both men and women.
- H2: Negative affect moderates the relationship between social media addiction and quality of life, such that higher negative affect accentuates the negative impact for both men and women.

### Proposed research model

The quality of life is a complex concept, defined in this study by five main dimensions [69]: material and physical well-being; relationships with other people; social, community, and civic activities; personal development and fulfillment; and recreation. Consistent with prior work and the theoretical rationale outlined in Theoretical background, the model posits a negative main effect of social media addiction on quality of life. The analysis examines these associations across the five dimensions using a multigroup SEM approach, testing gender-specific pathways and the moderating roles of positive and negative affect.

Building on these elements, the aim of this research is to explore the relationship between the “intensity” of the digitalization process, reflected by the use of social media, and the quality of life of Romanian adults. The model adopts a gender perspective (men and women) and tests the moderating effects of positive and negative affect. Thus, the title ‘Emoji or real emotions?’ emphasizes the study’s focus on the interplay between digitally mediated affect (expressed through social media interactions) and real-life emotional well-being.

**Fig. 1:**
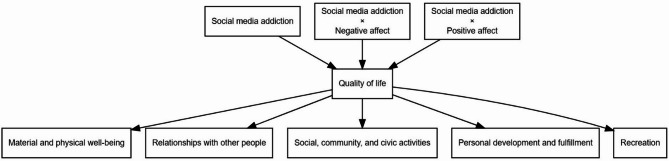
The hypothetical model

Figure 1 depicts the proposed multigroup model (social media addiction → quality of life), with positive affect (buffer) and negative affect (amplifier) specified as moderators.

## Method

### Study design

The current study, conducted in accordance with the Declaration of Helsinki (World Medical Association Helsinki declaration), was approved in August 2024 by the Ethics Committee (The Scientific Council of the University Research and Creation) from the West University of Timișoara, Romania.

The research has a cross-sectional survey design and data were collected between December 11, 2024 and February 8, 2025. All the participants in the study were informed about the context, objective and purpose of the study and provided with informed consent.

### Participants

The study was conducted with 217 participants (37.8% men and 62.2% women). The participants were Romanian citizens, adults, aged 18–62 years (M = 36.15, SD = 11.92). The study participants were high school graduates (24%), bachelor’s degree (35.9%), master’s degree (35%), and doctoral/postdoctoral degree (5.1%).

The target population for inference was Romanian adults who are digitally connected, active on social media, and currently enrolled in formal or non-formal education. We employed a non-probability convenience/volunteer online sampling strategy with purposive eligibility. Inclusion criteria were: (i) age ≥ 18; (ii) Romanian citizenship or residency; (iii) being digitally connected and active on social media (self-reported); (iv) enrolled in formal or non-formal education at or near the time of survey completion (self-reported); and (v) provision of informed consent. Exclusion criteria were: (i) age < 18 or lack of Romanian citizenship/residency; (ii) decline of consent; and (iii) substantially incomplete responses on primary study measures. No probability-based quotas were implemented.

This approach was selected because the focal constructs concern online behavior (social media use and addiction), probability sampling frames for Romanian online adults are not readily available and our goal was theory testing (SEM-based moderation analysis) rather than producing population estimates. To enhance coverage and heterogeneity, recruitment relied on non-overlapping online channels over a multi-week field period.

The sample size was calculated a priori with G*Power; for a medium effect size, power (1 − β) = 0.80, α = 0.05, and one predictor, the minimum required sample was *n* = 55.

The sample approximates the profile of digitally connected Romanian adults but over-represents urban residents (80.1%) and individuals engaged in formal/non-formal education. Accordingly, results should be generalized to the online adult population in Romania that meets the study’s eligibility criteria, rather than to all Romanian adults. Full demographics are reported to support assessments of external validity.

### Measures

The questionnaire addressed to the participants consisted of the following scales, as follows:

- *The Romanian version of the Social Media Addiction Scale*.

The Social Media Addiction Scale is a 29-question questionnaire with four components: virtual tolerance, virtual problems, virtual information, and virtual communication [13]. In the current study, the Romanian version of the Social Media Addiction Scale – Student Form, validated on the Romanian population by the authors of the paper [13], was used.

As described in the Introduction, the construct measured by this scale reflects problematic, maladaptive patterns of social media use consistent with contemporary frameworks of behavioral addiction [14, 15].

The Social Media Addiction Scale allows participants to record responses on a 5-point Likert scale (from 1-Strongly disagree, to 5-Strongly agree). In the current study, the Social Media Addiction Scale had very good internal consistency, with a Cronbach’s alpha value of 0.934.

b) *Positive and Negative Affect Scales*,* extended form - PANAS-X*.

The PANAS-X scales, consisting of 60 questions, measure General Positive Affect, General Negative Affect, as well as 11 specific affects: fear, sadness, guilt, hostility, shyness, fatigue, surprise, joviality, self-confidence, attention and calmness [70]. The feelings and emotions described by the PANAS-X scales refer to the period of the last weeks [71].

In the current study, only the following factors were used: General positive and negative affect, that is the general dimensions that describe the affective experience, for example: the extent to which a person feels excited, active or, on the contrary, aversive, guilty, fearful, nervous. The items were kept in the current research, according to the validated version of the scale, in Romanian [70].

The PANAS-X scales allow participants to record responses on a 5-option Likert scale (from 1- very slightly or not at all, to 5- extremely).

The 2 factors of the PANAS-X scale used in the current research had good internal consistency, with Cronbach’s alpha values: 0.891 (Negative Affect) and 0.843 (Positive Affect).

c) *The Quality of Life Scale – QOLS*.

The Quality of Life Scale was adapted by the study authors [69], after the original scale created by the American psychologist John Flanagan in the 1970s.

The QOLS scale consists of 16 items and is divided into 5 conceptual categories [69] or subscales [72]: Material and Physical Well-being (items 1, 2, 16), Relationships with other People (items 3, 4, 5, 6), Social, Community, and Civic Activities (items 7, 8), Personal Development and Fulfillment (items 9, 10, 11, 12) and Recreation (items 13, 14, 15).

The items allow participants to record responses on a 7-point Likert scale (from 1 = terrible to 7 = delighted).

As far as the current research is concerned, the QOLS tool had good internal consistency, with Cronbach’s alpha value: 0.915. In the SEM framework, the five QOLS domains were modeled as reflective indicators of a single latent quality of life construct. Therefore, reliability was assessed at the latent level using composite reliability (CR) and average variance extracted (AVE), rather than reporting separate Cronbach’s α coefficients for each domain. This approach is consistent with best practices in SEM, where construct-level reliability indices (e.g., CR, AVE) are preferred over reporting Cronbach’s α separately for subscales when domains are modeled as reflective indicators [73, 74].

The QOLS scale has been successfully used in numerous doctoral theses and research studies, conducted with Romanian participants, which attests to its suitability for this context, for example: [75–79].

No new translation was undertaken; we used the validated Romanian versions of the Social Media Addiction Scale and PANAS-X as cited, and the Romanian adaptation of QOLS; item wording followed the published versions.

### Procedure

Data collection was conducted online via the Google Forms platform. Before launching the survey, the study was pre-registered on the Open Science Framework platform (objectives, main hypotheses, study design, data collection procedure, measured variables and statistical analysis plan) at: https://osf.io/uhkrw/?view_only=007c92b8febb438e9c664f2b2a25d80a. It is also necessary to mention that the current study is a sub-study of the research on the theme: “The intensity of the digital age” and its impact on the quality of life – emotional and psychosocial perspective of the individual.

Recruitment was operationalized via non-overlapping online channels, email to distinct university mailing lists, independent professional networks, and separate public social-media groups (complemented by mobile-messaging posts to the same target groups), to broaden coverage while avoiding audience overlap. Each invitation briefly stated the study topic (social media use and well-being), eligibility, voluntariness, anonymity, and linked to the survey. On the landing page, respondents first viewed the information sheet and indicated informed consent via a required checkbox, after which a short screening block verified eligibility. Only eligible respondents then proceeded to complete the main study instruments (Social Media Addiction Scale, PANAS-X, and QOLS), along with demographic questions. To minimize duplicate participation, a one-response-per-participant safeguard was enabled, and submissions with substantial missingness on primary measures were excluded per the a priori rule described in § 3.2. No names or contact details were collected.

The general questionnaire was composed of demographic questions regarding the year of birth, gender, level of education, professional status, marital status, residential area (urban/rural), number of children, followed by: the Romanian version of the Social Media Addiction Scale, PANAS-X [71] and QOLS [69].

Because all instruments were validated Romanian versions (Social Media Addiction Scale; PANAS-X) or have been widely used with Romanian samples (QOLS), a separate psychometric pilot was not necessary. Prior to fieldwork, we conducted a procedural pretest of the Google Forms survey (consent flow, item rendering, skip/branch logic, device/browser compatibility, completion time). No changes to validated item wording were required; only interface and instructional clarifications were reviewed.

### Statistical analysis

**Fig. 2:**
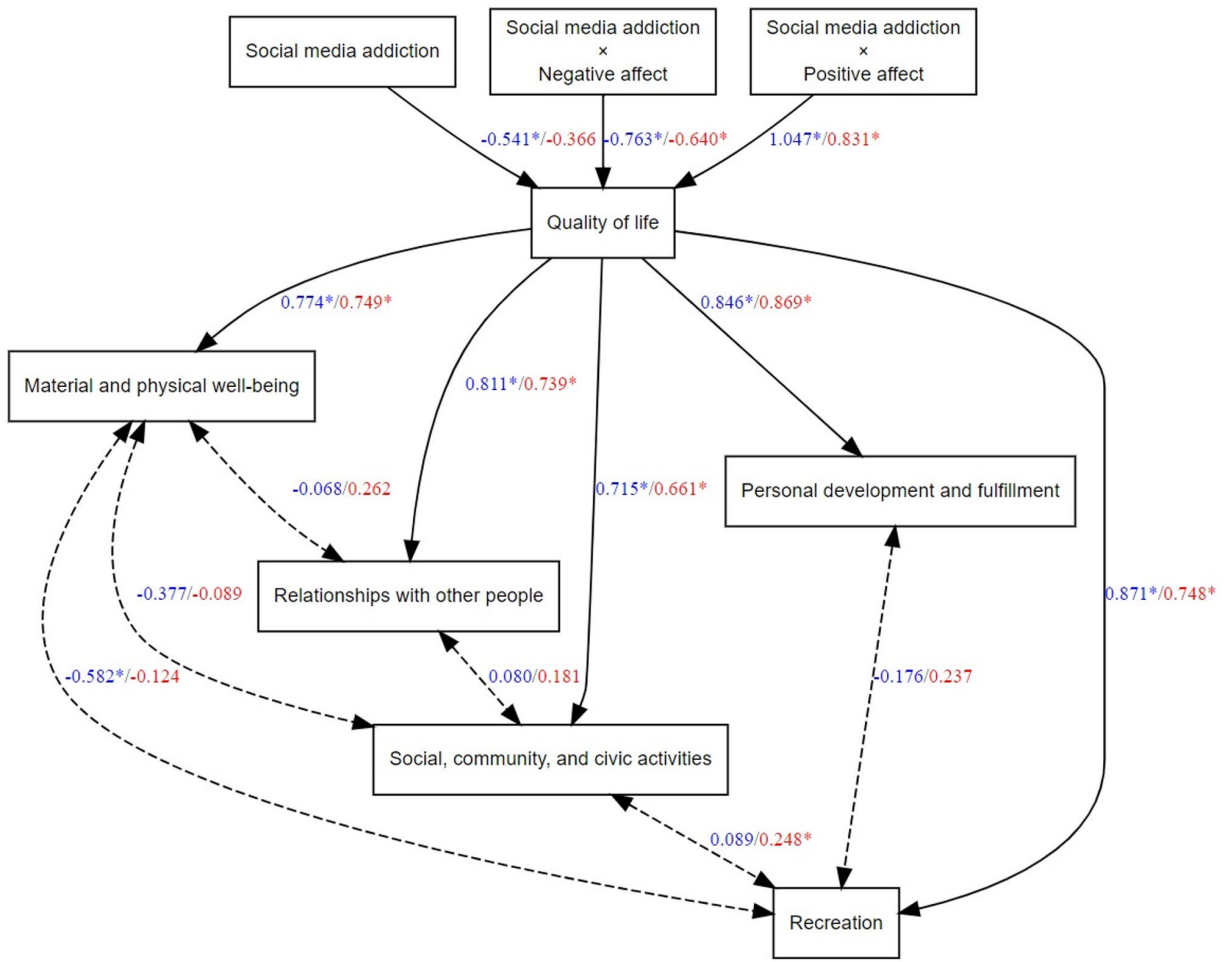
The hypothesized model (the group of men is represented by blue, and the group of women by red)

The statistical analysis was performed using the RStudio software - version 2024.12.0 (build 467) - Windows 10 Pro operating system, and the statistical method used was SEM. The lavaan package was used for the statistical analysis and the DiagrammeR, DiagrammeRsvg and rsvg packages were used for the graphical representation of the model (see Fig. 2).

The SEM analysis used in this work was of the multigroup type (the groups being separated by the gender variable: men/women). The analysis performed assumed the estimation of a single model for the two groups, without strict constraints, obtaining a single set of fit indices, but different values ​​of the regression coefficients per groups. The interpretation of the results was also performed separately for each group. Multigroup analysis is a set of techniques used when it is desired to evaluate differences between variables [80], such as gender or culture, using the same structural model [81].

With a view to evaluating the model proposed in this research (data-model fit), the criteria established in the paper [82] were used, namely: Comparative Fit Index (CFI) ≥ 0.96, Standardized Root Mean Square Residual (SRMR) ≤ 1.0, or Root Mean Square Error of Approximation (RMSEA) ≤ 0.06 and Standardized Root Mean Square Residual (SRMR) ≤ 0.08.

Invariance between groups, including partial invariance, was also assessed to ensure the correctness of the interpretations of the results (between groups). At the same time, the Simple Slope method was also used to assess the effects of the moderators at their different levels, thus clarifying how the relationship (social media addiction – quality of life) varies depending on the intensity of the moderators (positive affect and negative affect by gender) [83].

In an additional robustness model, we included age, urban residence (urban vs. rural), relationship status (in a couple vs. not), parental status (has children vs. none), and education (ordinal) as exogenous predictors of both the latent quality of life factor and the social media addiction predictor. Following best practice for interaction testing, positive and negative affect were also entered as main effects alongside the interaction terms.

Also, consistent with recommendations (e.g [84, 85]).,, we assessed potential common-method bias using Harman’s single-factor test and a one-factor CFA; model fit was evaluated with robust maximum likelihood (MLR) indices [86–88].

To verify whether assumptions for parametric analyses were met, we inspected univariate and multivariate normality, linearity, and homoscedasticity. Shapiro-Wilk tests [89] indicated slight deviations from normality for some variables; therefore, we computed Spearman’s rank-order correlations [90] alongside Pearson’s r to confirm robustness. The pattern of significant associations remained unchanged (e.g., social media addiction → quality of life: *r* = − 0.214, *p* < 0.001; ρ = −0.252, *p* < 0.001). Multivariate normality was assessed using Mardia’s test [91], which indicated significant deviations for the full sample (skewness = 299.73, *p* < 0.001; kurtosis = 5.48, *p* < 0.001) and within each group (men: skewness = 211.86, *p* < 0.001; kurtosis = 3.20, *p* < 0.001; women: skewness = 216.83, *p* < 0.001; kurtosis = 3.49, *p* < 0.001). To accommodate these deviations, SEM models were estimated using MLR [92], and robust fit indices are reported. Homoscedasticity was examined using the Breusch–Pagan test [93], which revealed no violations (men: BP = 0.005, *p* = 0.941; women: BP = 0.027, *p* = 0.871). Scatterplots further confirmed approximate linearity and absence of influential outliers. Overall, the analyses are rigorous and robust, fully supporting the validity and reliability of the reported results.

In all SEM analyses, quality of life was modeled as a latent construct defined by five observed indicators: material and physical well-being, relationships with other people, social/community/civic activities, personal development and fulfillment, and recreation. The total quality of life score was intentionally excluded from SEM analyses to avoid redundancy with its component subscales and to ensure a more accurate estimation of the latent construct.

## Results

### Common method bias

Procedurally, we minimized common-method bias through anonymity/voluntariness, varied response formats (5-point vs. 7-point), and placing demographics before the focal scales to create mild psychological separation [84]. We assessed potential common-method bias using two standard diagnostics. In a Harman single-factor check (unrotated one-factor solution), the first factor accounted for 49.3% of the variance (< 50%). In addition, a one-factor CFA with a single latent factor loading all indicators showed poor fit: χ²(20) = 76.03, *p* < 0.001; CFI = 0.92; TLI = 0.89; RMSEA = 0.12, 90% CI [0.09, 0.15]; SRMR = 0.054 (robust indices: CFI = 0.93; TLI = 0.90; RMSEA = 0.12). Taken together, these results indicate that a dominant common factor does not explain the observed relationships.

### Preliminary analysis

In Tables 1 and 2 we find the descriptive statistics and correlations between the study variables. All five quality of life subscales were retained as indicators in the SEM measurement model for men and women (see Table 3; Fig. 2). Analyzing Table 1, we can see that social media addiction is negatively and significantly correlated with three of the five factors of the quality of life, in the case of men: relationships with other people (*r* = −0.251, *p* < 0.05), personal development and fulfillment (*r* = −0.237, *p* < 0.05), recreation (*r* = −0.312, *p* < 0.01). In Table 2, we can see that social media addiction is negatively and significantly correlated with two of the five factors of the quality of life, in the case of women: material and physical well-being (*r* = −0.172, *p* < 0.05) and relationships with other people (*r* = −0.189, *p* < 0.05). These correlations support the main idea of ​​the current study: the excessive use of social media is associated with a lower quality of life.

Considering the relationship between the moderators and the predictor, we can notice that, in the case of both men and women, the positive affect moderator is not significantly correlated with social media addiction. Regarding the correlation between the negative affect moderator and social media addiction, there are significant positive correlations for both groups in the research, with the difference that, in the case of men, the correlation is moderate (*r* = 0.442, *p* < 0.001) and in the case of women, the correlation is weak (*r* = 0.318, *p* < 0.001). These results highlight the fact that the proposed moderation relationships are methodologically valid.

**Table 1:** Descriptive statistics and correlations between the investigated variables (Group: Men)

	Mean	SD	1	2	3	4	5	6	7	8	9
1. Social media addiction	57.28	20.001	—								
2. Positive affect	32.622	7.456	−0.116	—							
3. Negative Affect	19.268	7.435	0.442***	−0.214	—						
4. Material and physical well-being	17.085	3.21	−0.214	0.42***	−0.564***	—					
5. Relationships with other people	20.829	4.939	−0.251*	0.608***	−0.48***	0.611***	—				
6. Social, community, and civic activities	9.585	2.712	−0.177	0.457***	−0.42***	0.36***	0.614***	—			
7. Personal development and fulfillment	21.22	5.448	−0.237*	0.654***	−0.516***	0.648***	0.741***	0.63***	—		
8. Recreation	16.049	3.823	−0.312**	0.559***	−0.513***	0.474***	0.709***	0.638***	0.726***	—	
9. Age	30.512	10.13	−0.243*	0.261*	−0.331**	0.412***	0.346**	0.346**	0.408***	0.366***	—

* p < 0.05, ** p < 0.01, *** p < 0.001, *SD * Standard Deviation, 1= Social media addiction, 2 = Positive affect, 3 = Negative Affect, 4 = Material and physical well-being, 5 = Relationships with other people, 6 = Social, community, and civic activities, 7 = Personal development and fulfillment, 8 = Recreation, 9 = Age

**Table 2:** Descriptive statistics and correlations between the investigated variables (Group: Women)

	Mean	SD	1	2	3	4	5	6	7	8	9
1. Social media addiction	56.593	19.022	—								
2. Positive affect	33.726	7.11	0.042	—							
3. Negative Affect	21.889	8.287	0.318***	−0.403***	—						
4. Material and physical well-being	16.867	3.176	−0.172*	0.514***	−0.605***	—					
5. Relationships with other people	21.6	4.686	−0.189*	0.476***	−0.532***	0.671***	—				
6. Social, community, and civic activities	10.133	2.807	−0.028	0.524***	−0.424***	0.482***	0.6***	—			
7. Personal development and fulfillment	22.711	4.317	−0.158	0.577***	−0.513***	0.648***	0.613***	0.602***	—		
8. Recreation	16.044	4.263	−0.11	0.504***	−0.494***	0.536***	0.586***	0.647***	0.741***	—	
9. Age	39.578	11.65	−0.295***	0.052	−0.217*	0.045	0.094	0.064	0.128	0.007	—

* p < 0.05, ** p < 0.01, *** p < 0.001, *SD* Standard Deviation, 1 = Social media addiction, 2 = Positive affect, 3 = Negative Affect, 4 = Material and physical well-being, 5 = Relationships with other people, 6 = Social, community, and civic activities, 7 = Personal development and fulfillment, 8 = Recreation, 9 = Age

CR and AVE for the latent quality of life factor indicated adequate convergent validity (CR ≥ 0.70; AVE ≥ 0.50) in both groups: women, CR = 0.874, AVE = 0.581; men, CR = 0.900, AVE = 0.643. The square root of AVE for quality of life (0.76 in women; 0.80 in men) reflects strong indicator loadings. Because only one latent construct was modeled, a Fornell–Larcker discriminant-validity comparison is not applicable.

### The relationship between social media addiction and the quality of life, moderated by positive affect and negative affect – multigroup SEM analysis

The test indicated a good suitability of the model, according to the criteria [82], and the theoretical specification of the relationships between the variables is adequate: CFI = 0.987, SRMR = 0.043, RMSEA (scaled) = 0.052, 90% CI [0.000, 0.096]. Also, CMINF/DF = 1.367, NFI = 0.956, P-value (Chi-square) = 0.143 (scaled df = 26), TLI (scaled) = 0.976, indicate a good suitability.

In order to check the invariance of the model, a fully constrained model was initially tested (equalizing the loading factors and intercepts between groups). The results suggested the lack of strict invariance, indicating an increase in RMSEA above 0.08 (from 0.052 to 0.086). The chi-square (*p* = 0.003) became significant, suggesting differences between groups, and in addition, the comparison of SEM coefficients between groups showed significant variations (e.g., social media addiction → quality of life: insignificant value for the women’s group (*p* = 0.1), while for the men’s group the value was significant (*p* = 0.006)). These results also highlighted the fact that negative and positive affect have very different effects between groups on the quality of life, which justified the need for a more flexible approach.

Therefore, a partial invariance approach was applied, relaxing the constraints for the coefficients associated with negative and positive affect. The final model showed a good fit to the data (RMSEA = 0.052, CFI = 0.987, TLI = 0.976, SRMR = 0.043), which allows comparing the results between groups with caution, given that the model is not completely invariant.

Moreover, it is necessary to mention the fact that within the current model, we allowed the following covariances, supported, on the one hand, by the initial covariance matrix of each group (men – Table 1 and women – Table 2), where these variables present significant correlations, and on the other hand, by the modification indices – MI which indicated that these covariances are necessary to maintain the optimal structure of the model:

- pesonal development and fulfillment ~ ~ recreation (correlation matrices: men’s group: r*r* = 0.726, *p* < 0.001; women’s group: *r* = 0.741, *p* < 0.001; MI = 6.016). The relationship is also supported by the specialized literature, recreational activities can support personal development from a psychological, sporting, cultural, etc. point of view [94, 95].
- material and physical well-being ~ ~ relationships with other people (correlation matrices: men’s group: *r* = 0.611, *p* < 0.001; women’s group: *r* = 0.671, *p* < 0.001; MI = 8.530). According to the literature, the importance of social relationships is demonstrated by their impact on the general functioning of the person and on their physical health [96].
- social, community and civic activities ~ ~ recreation (correlation matrices: men’s group: *r* = 0.638, *p* < 0.001; women’s group: *r* = 0.647, *p* < 0.001; MI = 7.347). Social activities can be combined with recreational activities, for example, visiting family and friends, playing team sports, learning new skills such as dancing, art classes, tourism, etc [97, 98].
- relationships with other people ~ ~ social, community and civic activities (correlation matrices: men’s group: *r* = 0.614, *p* < 0.001; women’s group: *r* = 0.6, *p* < 0.001; MI = 5.620). The specialized literature confirms the link between the two factors, for example, social participation is a predictor of interpersonal relationships [99].
- material and physical well-being ~ ~ recreation (correlation matrices: men’s group: *r* = 0.474, *p* < 0.001; women’s group: *r* = 0.536, *p* < 0.001; MI = 8.823). The relationship is also supported by the specialized literature: the access to financial resources increases recreational opportunities, for example, through access to travel, participation in cultural activities [100].
- material and physical well-being ~ ~ social, community and civic activities (correlation matrices: men’s group: *r* = 0.36, *p* < 0.001; women’s group: *r* = 0.482, *p* < 0.001; MI = 9.212). The specialized literature confirms the connection between the two factors, for example material well-being influences the level of civic involvement [101].

In order to assess the impact of covariances on model suitability, MI was calculated initially on the original model and subsequently on the partial invariance model. The MI analysis showed that certain covariances are necessary to maintain an optimal model structure. In the partial invariance model, two covariances were confirmed as relevant: personal development and fulfillment ~ ~ recreation and relationships with others ~ ~ social, community, and civic activities. The remaining covariances were initially identified on the original model and maintained in the final model due to the empirical support provided by the correlation matrix and the MI index.

The quality of life is included in the model as a latent variable, defined by five dimensions: material and physical well-being; relationships with other people; social, community and civic activities; personal development and fulfillment, and recreation. All of these dimensions have high standardized loadings (≥ 0.66 for women and ≥ 0.71 for men), indicating that they are relevant in defining the quality of life for both groups – see Fig. 2; Table 3. In Fig. 2, the values ​​associated with the male group are written in blue while red was used for women.

**Table 3:** Standardized loadings (β) for the latent quality of life construct by gender

Quality of Life–- dimensions	β	*p*
Material and physical well-being–- Men	0.774	< 0.001
Material and physical well-being–- Women	0.749	< 0.001
Relationships with other people–- Men	0.811	< 0.001
Relationships with other people–- Women	0.739	< 0.001
Social, community, and civic activities–- Men	0.715	< 0.001
Social, community, and civic activities–- Women	0.661	< 0.001
Personal development and fulfillment–- Men	0.846	< 0.001
Personal development and fulfillment–- Women	0.869	< 0.001
Recreation–- Men	0.871	< 0.001
Recreation–- Women	0.748	< 0.001

β Standardized loadings, *p* p-value

As we can see in Fig. 2, in the case of the male group, material and physical well-being is negatively correlated with recreation (β = −0.582, *p* < 0.05), which may indicate that people who relax more do not necessarily report a high state of well-being. Also, in the case of the female group, we find the relationship social, community and civic activities – recreation as significant (β = 0.248, *p* < 0.05), suggesting that participation in social activities may be a factor of relaxation.

As to the direct effects, in relation to the hypotheses of this study, the following results were obtained: social media addiction predicts a decrease in the quality of life only in men (β = −0.541, *p* < 0.01). In the case of women, the relationship is not significant.

The interaction effect between social media addiction and negative affect is significant for both men (β = −0.763, *p* < 0.001) and women (β = −0.640, *p* < 0.001). This result shows that when social media addiction is accompanied by negative affect, the quality of life decreases significantly for both groups, but the effect is stronger for men.

The interaction effect between social media addiction and positive affect is also significant for both men (β = 1.047, *p* < 0.001) and women (β = 0.831, *p* < 0.001). Positive affect moderates the relationship between social media addiction and the quality of life in a positive sense. In other words, for those who have a high level of positive affect, the negative impact of social media addiction on the quality of life is compensated, and this effect is stronger for men.

Therefore, the two hypotheses (H1, H2) of the current study were confirmed by the statistical analysis performed in both groups evaluated (men, women): positive affect as a moderator of the relationship between social media addiction and the quality of life has a protective role in both groups, but stronger in the case of men, while the negative affect moderator has a significant negative effect on both groups, but the effect is also stronger in the case of men.

The proposed model explains well the variance of the quality of life for both groups, but better for the male group (male group: R² = 0.617; female group: R² = 0.561). The results obtained indicate that 61.7% of the variance of the quality of life is explained by the model in the case of men, and 56.1% in the case of women. The SEM models that explain over 50% of the variance of a latent variable are considered moderate to strong/substantial [102].

Considering the analysis of the relationship (social media addiction → quality of life) on various levels of moderators (positive affect and negative affect), the Simple Slopes Analysis technique was used in lavaan - the level moderation test and the following results were obtained:

- positive affect moderator:

In an additional covariate-augmented multigroup SEM (age, urban residence, relationship status, parental status, education), with positive and negative affect entered as main effects alongside the interactions, the substantive pattern held: positive affect was positively and negative affect negatively associated with quality of life in both groups; the direct social media addiction→quality of life path attenuated and was non-significant; and the interaction terms were also non-significant under this conservative specification. These checks suggest that demographics do not drive the affect–quality of life links; the moderation estimates are sensitive to model specification.

These results partially reconfirm the validity of H1 hypothesis.

b) negative affect moderator:

In the case of men, the moderator effect of negative affect in the relationship between social media addiction and the quality of life is significant (p < 0.05) regardless of the level. As negative affect increases, the negative impact of social media addiction on the quality of life becomes stronger (−1SD: b = −0.067, 95% CI [−0.121, −0.014], p < 0.05; SD: b = −0.082, 95% CI [−0.137, −0.026], p < 0.01; +1SD: b = −0.096, 95% CI [−0.154,−0.038], p < 0.01). The values ​​obtained for each level of the negative affect moderator are significant, given that the confidence intervals do not contain the value 0. 

In the case of the group formed by women, the effect of negative affect moderator intervened in the relationship between social media addiction and the quality of life is similar in direction to the effect obtained in the case of men, but much stronger (*p* < 0.001), for all levels of the moderator. This means that women are more affected by the effects of social media addiction than men, especially those with high negative affect: the negative effect increases as negative affect increases (−1SD: b = −0.104, 95% CI [−0.157, −0.050], *p* < 0.001; SD: b = −0.125, 95% CI [−0.181, −0.068], *p* < 0.001; +1SD: b = −0.146, 95% CI [−0.206, −0.085], *p* < 0.001). Also, the confidence intervals for each level of the moderator do not contain the value 0, which means that they are significant.

These results, in turn, reconfirm the validity of H2 hypothesis.

In an additional covariate-augmented multigroup SEM (age, urban residence, relationship status, parental status, education), with positive and negative affect entered as main effects alongside the interactions, the substantive pattern held: positive affect was positively and negative affect negatively associated with quality of life in both groups; the direct social media addiction→quality of life path attenuated and was non-significant; and the interaction terms were also non-significant under this conservative specification. These checks suggest that demographics do not drive the affect–quality of life links; the moderation estimates are sensitive to model specification.

## Discussions

Social media addiction is manifested by a compulsion to use them excessively, and their prolonged use is associated, in the specialized literature, with mental health problems and with the impairment of the individuals’ well-being [103, 104]. In this context, the aim of the current study was to analyze the relationship between social media addiction and the quality of life, on a sample of 217 adult Romanian citizens, taking into account the positive affect and negative affect moderators, at the group level: men and women.

The descriptive statistical analysis (see Tables 1 and 2) allows us to understand the gender differences regarding the impact of social media on personal and social quality of life. Significant correlations with weak and moderate values ​​were recorded between the main variables of the study, supporting the research hypotheses. Social media addiction is associated with a decrease in material and physical well-being only for women (*r* = −0.172, *p* < 0.05). In the specialized literature, we find that women are more affected by social pressure and comparison on social media, which can contribute to financial stress and negative body image [105–107]. On the other hand, especially in the male group, social media addiction is negatively correlated with social relationships (*r* = −0.251, *p* < 0.05), compared to the female group (*r* = −0.189, *p* < 0.05). The literature also confirms these relationships: previous studies show that men are more likely to reduce the time spent in face-to-face social interactions (offline) in favor of digital activities, such as the excessive use of social media, gaming or just consuming online content [108, 109].

From the descriptive statistics, we can also notice that social media addiction affects personal development (*r* = −0.237, *p* < 0.05) and recreation (*r* = −0.312, *p* < 0.01) to a greater extent for men than for women, indicating that the excessive use of social media can interfere with hobbies and self-development activities.

If we refer to age, we can infer from Tables 1 and 2 that social media addiction decreases significantly with age, in both groups, men and women, but more in the case of women (women: *r* = −0.295, *p* < 0.001 versus men: *r* = −0.243, *p* < 0.05). The result is not surprising, given that young people are more vulnerable to social media addiction, often without the ability to correctly assess the harmful consequences of this behavior [110].

The quality of life is included in the model as a latent variable, having as dimensions: material and physical well-being; relationships with other people; social, community and civic activities; personal development and fulfillment; and recreation. Although all the standardized loading factors of the quality of life dimensions are high (β ≥ 0.66, *p* < 0.001) for both groups, it is necessary to state that the dimension of personal development and fulfillment has the highest loading for women (β = 0.869, *p* < 0.001), which suggests that personal development is the most important predictor of the quality of life for them. In contrast, for men, recreation (β = 0.871, *p* < 0.001) along with relationships with other people have the highest loadings (β = 0.811, *p* < 0.001). These results indicate that relaxation along with relational satisfaction have an important role in determining the quality of life for men. The results obtained are supported by the specialized literature, both for men and women.

Considering the results obtained by gender, the analysis is consistent with studies suggesting that men tend to use social media more for building new relationships [111] and consuming content [112], and addiction may lead to a higher decrease in life satisfaction (β = −0.541, *p* < 0.05) [113].

Although positive affect influences positively the quality of life in both groups, the standardized coefficient indicates that women benefit from the protective effect of positive emotions to a lesser extent. This can be explained by the fact that women are more inclined to use social media for social connection and emotional support [114, 115], which helps them mitigate the negative effects of excessive use.

Analyzing the significant correlations between the quality of life dimensions (see also Fig. 2), we can notice that in the case of the group formed by men, there is a significant correlation between material and physical well-being, and recreation (β = −0.582, *p* < 0.05), which indicates that when men feel good, they tend to be more active and implicitly less physically relaxed. Although it seems a contradiction, we find clarifications again in the specialized literature, where studies show that men are more active than women in their free time, for example, they get involved in team sports [116–118]. In their turn, women positively associate social, community and civic activities with recreation (β = 0.248, *p* < 0.05), a situation which is not all surprising, confirmed by previous studies, taking into account that women are more inclined towards socializing, including as a relaxation activity [50, 119].

## Theoretical implications

This section synthesizes the study’s theoretical implications. The findings indicate that the relationship between social media addiction and quality of life is contingent on affective states (positive/negative affect) and differs by gender, thereby extending moderation-based accounts of social media addiction → quality of life and clarifying when and for whom adverse effects are most pronounced.

As far as the proposed moderators of the model positive affect and negative affect are concerned, we can infer from the descriptive statistics that they have strong correlations with the quality of life dimensions in both groups, with the difference that, for positive affect, the correlations are positive, and for negative affect, the correlations are negative. We can also infer from the descriptive statistics that positive affect is not significantly correlated with social media addiction (predictor), while negative affect is significantly correlated, weakly to moderately, with social media addiction in both groups (*r* = 0.318, *p* < 0.001 for the women’s group and *r* = 0.442, *p* < 0.001 for the men’s group).

These results indicate that emotional affect strongly influences the quality of life, which means that it can be a moderating variable (either attenuating or amplifying the negative effects of social media addiction). Interpreted through UGT, these patterns are consistent with need-driven engagement that can displace offline activities central to quality of life; affective states condition whether such displacement translates into diminished functioning or is buffered by positive emotional resources. All these results are also found in the specialized literature, for example, positive affect can help individuals adjust themselves in stressful situations [120], while negative affect worsens the perception of stress [121], including stress generated by social media addiction.

The H1 Hypothesis of the analyzed model, which aimed to test the relationship between social media addiction and the quality of life, under the influence of positive affect as a moderator, was confirmed for both analyzed groups: men and women. Positive affect has a significant protective effect on the quality of life, in relation to social media addiction, in both groups (men’s group: β = 1.047, *p* < 0.001; women’s group: β = 0.831, *p* < 0.001). Compared to the direct relationship: social media addiction – quality of life (male group: β = −0.541, *p* < 0.05; female group: β = −0.366, *p* = 0.097), the significant effect of positive affect on the quality of life can be noticed, especially in the case of men (from β = −0.541, *p* < 0.05 to β = 1.047, *p* < 0.001). In the case of the group consisting of women, where social media addiction does not have a significant direct impact on the quality of life, (*p* = 0.097), positive affect still has a significant role in increasing the quality of life, suggesting an attenuation of other possible negative effects.

The results of the proposed model align with the specialized literature, highlighting that positive affect contributes to an improved perception of the quality of life [33, 122], regardless of the negative external influences, in this case, excessive use of social media. On the other hand, the experience of positive emotions broadens the momentary thought-action patterns [33], which could explain why the users with a high level of positive affect do not feel so strongly the negative effects of social media addiction on the quality of life. This pattern accords with Broaden-and-Build logic (broadened thought–action repertoires and accrued resources) and contrasts with Conservation of Resources views of resource loss spirals, wherein heightened negative affect exacerbates strain and undermines QoL [34].

These findings can also be interpreted through the lens of compulsive emotion regulation in social media use. Prior research suggests that online social networking can temporarily fulfill emotional needs and enhance positive affect [123]. Building on this, our results indicate that such short-term enhancement may come at the cost of reduced self-awareness and maladaptive coping strategies, as excessive engagement can foster compulsive use and ultimately diminish well-being in the long term. Consistent with this perspective, we found that while positive affect appears to buffer the negative association between social media addiction and quality of life, high negative affect amplifies this harmful link, suggesting that individuals experiencing distress may be more vulnerable to using social media as an emotion regulation tool.

The H2 hypothesis of the model aimed to test the relationship between social media addiction and the quality of life, under the influence of negative affect as a moderator, was confirmed, in its turn, for both groups analyzed: men and women. Negative affect significantly decreases the quality of life in both groups, men and women, but the effect is stronger in the case of men (men’s group: β = −0.763, *p* < 0.001 versus women’s group: β = −0.640, *p* < 0.001).

In the men’s group, where social media addiction already has a significant negative effect on the quality of life (β = −0.541, *p* < 0.05), the negative affect amplifies this effect. In the case women, although social media addiction is not a significant predictor of the quality of life (β = −0.366, *p* = 0.097), negative affect has a clear negative impact, suggesting that other sources of stress may play an important role. Negative affect is associated with more pessimistic perceptions of life [65, 124] and a reduced capacity for emotional adjustment or the use of less effective coping strategies [66, 125], which may explain why individuals with high levels of negative affect are more vulnerable to the harmful influences of social media addiction. Also, negative affect is closely linked to increased stress, depression and anxiety [67, 68, 126], in this case also induced by the intense use of social media, especially through social comparison [127, 128] and exposure to negative content [46].

Considering the results obtained by gender, the analysis highlights that negative affect (negative emotional states such as anxiety and depression, for example) has a stronger influence on the perception of the quality of life for men than for women. The literature shows that men often have fewer emotional coping mechanisms [47, 48], compared to women, which may explain this increased vulnerability. Women, in their turn, have a higher degree of reflexivity [49], tending to examine their emotional experiences before acting, while choosing more effective coping strategies. Taken together, the moderation magnitudes align with the gender-based rationale outlined in Affect mechanisms and gender rationale regarding differences in emotion processing and regulation, clarifying why the buffering by positive affect and the amplification by negative affect vary across groups.

As for men, the explanation can be found in the PERMA theory [32], where it is mentioned that involvement in enjoyable activities and social connections are important well-being factors. In the case of women, professional achievement, personal success and generally speaking, their determination to develop successful careers, are already well-known elements [129–131].

Assessing the influence of the positive affect moderator across levels in the relationship between social media addiction and the quality of life, we find out that both men and women with a high level of positive affect may be less influenced by the negative consequences of social media addiction. This result, as previously mentioned, is supported by the theory [33] on the positive role of emotions in adaptability, according to which positive emotions broaden the cognitive and behavioral perspective, facilitating better management of experiences and stressors. Regarding the negative affect moderator, for both groups, men and women, the effect of social media addiction intensifies as the level of the moderator increases (from below average to above average), but in the case of women, the increase is steeper. This finding is confirmed by previous research on higher sensitivity in perceiving emotions in women [51, 52], while men tend to adopt more active coping strategies [132, 133], becoming less sensitive to external emotional influences.

Theoretically, these results (a) integrate UGT with affect-based mechanisms to specify conditions under which social media addiction relates to quality of life, (b) extend Broaden-and-Build and stress/affect perspectives by demonstrating dual moderation in a multigroup SEM, and (c) add gender-differentiated evidence from an underrepresented context. By articulating when (affect levels) and for whom (gender) the social media addiction → quality of life association strengthens or weakens, the study refines existing frameworks and advances a more conditional, mechanism-focused understanding of digital engagement and well-being.

## Practical implications

The results highlight that the impact of social media is not uniform, but differs according to emotional regulation and gender differences, which has important implications for future interventions. For example, the strategies to reduce the negative effects of social media addiction should focus on increasing positive affect. Also, mental health interventions should specifically target people with high levels of negative affect, through cognitive-behavioral psychotherapy programs and social support.

These recommendations are supported by the results of our multigroup SEM analysis, which explained 61.7% of the variance in quality of life for men and 56.1% for women (R² = 0.617; R² = 0.561). For example, the moderating effect of positive affect (β = 1.047, *p* < 0.001 for men; β = 0.831, *p* < 0.001 for women) suggests that interventions focused on enhancing positive emotional experiences — such as mindfulness training, resilience-building programs, and positive psychology techniques — can significantly mitigate the negative impact of social media addiction.

Conversely, negative affect showed a strong exacerbating effect on the relationship between social media addiction and quality of life (β = −0.763, *p* < 0.001 for men; β = −0.640, *p* < 0.001 for women), suggesting that psychological counseling and cognitive-behavioral therapy (CBT) should prioritize individuals with high levels of anxiety, stress, or depressive symptoms. Moreover, incorporating emotional regulation modules into school curricula or workplace well-being programs could reduce vulnerability to the negative consequences of intensive social media use. In addition, gender differences suggest the need for a personalized approach.

Finally, our findings emphasize the need for gender-sensitive interventions. Since men in our study reported stronger associations between social media addiction and decreased quality of life, while women exhibited higher vulnerability to negative affect, policymakers and practitioners should tailor prevention and intervention strategies accordingly. For example, male-oriented programs could focus on reducing excessive digital engagement and promoting offline social activities, while female-oriented programs might target emotional coping mechanisms and support networks.

## Limitations and future research recommendations

Although the current descriptive, exploratory, differential and correlational research makes a significant contribution to the specialized literature, it is constrained by a number of limitations. Firstly, the current study has a cross-sectional design and therefore does not provide the possibility to identify causal inferences [134]. For more accuracy and certainty in the results, it is desirable that future studies adopt a longitudinal design.

The study is based on self-administered questionnaires, which is another limitation, given the possibility that participants may not have provided truthful answers, which could lead to distorted study conclusions [135]. Therefore, future studies could use neurophysiological measurement tools, instead of questionnaires, for higher accuracy in the final results.

This study used a non-probability online sample of Romanian adults, which is appropriate for theory testing but limits population inference. The urban and education-engaged profile indicates potential over-representation of digitally connected, higher-educated respondents; therefore, findings are most applicable to the online adult population rather than all Romanian adults. Future work should consider probability sampling or post-stratification weighting to align with national benchmarks (e.g., region, urbanicity, age, education) and extend recruitment to rural and older populations. Replication with probability-based designs would strengthen external validity.

Future studies may also consider exploring intergenerational differences in research similar to the present one in order to identify specific patterns of social media use and their effects on the quality of life. In addition, future research should integrate other relevant psychological factors into the model to explain variance in the quality of life more accurately. For example, variables such as self-esteem, emotion regulation strategies, perceived social support, loneliness, resilience, and fear of missing out (FoMO) could provide a more nuanced understanding of how individual differences shape the relationship between social media use and well-being [136–138].

## Conclusion

This study provides valuable insights into how social media addiction affects the quality of life among Romanian adults, highlighting the moderating roles of positive and negative affect and the importance of gender differences. The findings demonstrate that positive affect has a protective effect, while negative affect amplifies the negative impact of social media addiction on quality of life. These results contribute to the broader theoretical understanding of emotional regulation in the digital “era” and emphasize the need for personalized strategies in addressing social media addiction. Practical implications include designing interventions that enhance positive affect and provide targeted support for individuals with high negative affect, particularly considering gender-specific patterns. Despite its limitations, this study fills an important gap in the literature by examining a culturally underrepresented population and offers a foundation for future research exploring digital behavior and well-being.

## Data Availability

The original contributions presented in this study are included in this article, and further inquiries can be directed to the corresponding author.

## Abbreviations

1 − β: Statistical power
AD: Anno Domini
AVE: Average Variance Extracted
α (alpha): Significance level
b: Unstandardized regression coefficient
BP: Breusch-Pagan
β (beta): Standardized regression/path coefficient
CBT: Cognitive-Behavioral Therapy
CFA: Confirmatory Factor Analysis
CFI: Comparative Fit Index
CI: Confidence interval
CR: Composite reliability
χ² (CMIN/DF): Chi-square / degrees-of-freedom ratio
FoMO: Fear of Missing Out
H: Hypothesis
M: Mean
MI: Modification indices
MLR: Maximum Likelihood Robust
n: Sample size
NFI: Normed Fit Index
p: p-value
PANAS-X: Positive and Negative Affect Schedule – Expanded Form
QOLS: Quality of Life Scale
r: Pearson correlation coefficient
R²: Coefficient of determination
ρ (rho): Spearman’s rho
RMSEA: Root Mean Square Error of Approximation
SD: Standard deviation
SDT: Self-Determination Theory
SEM: Structural equation modeling
SRMR: Standardized Root Mean Square Residual
TLI: Tucker–Lewis Index
UGT: Uses and Gratifications Theory

## References

[1] Donati JC. The Greek agora in its Peloponnesian context(s). Classical archaeology in context. De Gruyter; 2015. pp. 177–218. 10.1515/9781934078471-011.

[2] Gorski GJ, Packer JE. The Roman forum: A reconstruction and architectural guide. Cambridge University Press; 2015.

[3] Giffard CA. Ancient Rome’s daily gazette. Journalism Hist. 1975;2(4):106–32. 10.1080/00947679.1975.12066791.

[4] Huelsen C. The Roman forum, its history and its monuments. 2nd ed. Benedict C, translator. G. E. Stechert & Company; 1909.

[5] Davis JL. Social media. The international encyclopedia of political communication. Wiley; 2016. pp. 1–8. 10.1002/9781118541555.wbiepc004.

[6] Codrean R. A debate on the role of social media in business communication. MASTERCOM Politehnica Graduate Student J Communication. 2021;6(1):107-114. https://pgsj.upt.ro/current/item/78-a-debate-on-the-role-of-social-media-in-business-communication

[7] DataReportal WA. Social & Meltwater. Main reasons for using social media in Romania in 2024. Statista. 2024. https://www.statista.com/statistics/1378973/romania-main-reasons-for-using-social-media/

[8] Duong CTP. Social media. A literature review. J Media Res. 2020;13(3):112–26. 10.24193/jmr.38.7.

[9] DataReportal M, We Are Social. &. Number of internet and social media users worldwide as of February 2025 (in billions). Statista. 2025. https://www.statista.com/statistics/617136/digital-population-worldwide/

[10] Shaikh TA. Understanding the emergence and impact of social media platforms. IOSR J Humanit Social Sci. 2024;29(4):26–30. 10.9790/0837-2904102630.

[11] Population Reference Bureau. Total population of EU member States in 2024 with a forecast for 2050, by country (in million inhabitants). Statista. 2024. https://www.statista.com/statistics/253383/total-population-of-the-eu-member-states-by-country/

[12] Statista Research Department. Social media usage in Romania – statistics & facts. Statista. 2024. https://0610zz0ck-y-https-www-statista-com.z.e-nformation.ro/topics/7134/social-media-usage-in-romania/#topicOverview

[13] Ursoniu S, Serban CL, Giurgi-Oncu C, Rivis IA, Bucur A, Papava I, et al. Validation of the Romanian version of the social media addiction Scale-Student form (SMAS-SF) among undergraduate medical students. Neuropsychiatr Dis Treat. 2022;18:1195–205. 10.2147/NDT.S368476. 35734548 10.2147/NDT.S368476 PMC9207124

[14] Griffiths M. A ‘components’ model of addiction within a biopsychosocial framework. J Subst Use. 2005;10(4):191–7. 10.1080/14659890500114359.

[15] Kuss D, Griffiths M. Social networking sites and addiction: ten lessons learned. Int J Environ Res Public Health. 2017;14(3):311. 10.3390/ijerph14030311. 28304359 10.3390/ijerph14030311 PMC5369147

[16] Reuters Institute for the Study of Journalism. Most used social media and messaging platforms for news consumption in Romania in 2024. Statista. 2024. https://www.statista.com/statistics/1198565/romania-social-media-platform-for-news-consumption/

[17] Shin H, Park C. Gender differences in social networks and physical and mental health: are social relationships more health protective in women than in men? Front Psychol. 2023;14:1216032. 10.3389/fpsyg.2023.1216032. 38213610 10.3389/fpsyg.2023.1216032 PMC10782512

[18] Fashami AM. Gender differences in the use of social media: Australian postgraduate students’ evidence. Int J Social Sci Hum Res. 2020;3(12). 10.47191/ijsshr/v3-i12-03.

[19] Mazman G, Usluel YK. Gender differences in using social networks. Turkish J Educational Tech. 2011;10(2):133-139 https://www.researchgate.net/publication/279622067

[20] Hossain MS, Prodhan R. Gender difference of social media sites usage and its effects on academic performance among university students in Bangladesh. Eur Mod Stud J. 2020;4(5):121-130 https://journal-ems.com/index.php/emsj/article/view/134/128

[21] Theophilou E, Hernández-Leo D, Gómez V. Gender‐based learning and behavioural differences in an educational social media platform. J Comput Assist Learn. 2024;40(6):2544–57. 10.1111/jcal.12927.

[22] Katz E, Blumler JG, Gurevitch M. Uses and gratifications research. Public Opin Q. 1974;14(3):419–42. 10.1086/268109.

[23] Masrom M, Busalim A, Griffiths MD, Asadi S, Mohd Ali R. The impact of excessive Instagram use on students’ academic study: a two-stage SEM and artificial neural network approach. Interact Learn Environ. 2023;32(7):1–20. 10.1080/10494820.2023.2184393.

[24] Bhatiasevi V. The uses and gratifications of social media and their impact on social relationships and psychological well-being. Front Psychiatry. 2024;15:1260565. 10.3389/fpsyt.2024.1260565. 38501079 10.3389/fpsyt.2024.1260565 PMC10944947

[25] Hajdarmataj F, Paksoy AF. Uses andgratifications theory in social media applications: today’s active users, characteristics andobtained gratifications. In: Yüüksel E, Paksoy AF, Cingi CC, Durul SS, editors. Current Studies inCommunication Sciences-1. Literatüürk Academia. 2022.;24–35.

[26] Hossain MA. Effects of uses and gratifications on social media use. PSU Res Rev. 2019;3(1):16–28. 10.1108/PRR-07-2018-0023.

[27] Falgoust G, Winterlind E, Moon P, Parker A, Zinzow H, Chalil Madathil K. Applying the uses and gratifications theory to identify motivational factors behind young adult’s participation in viral social media challenges on TikTok. Hum Factors Healthc. 2022;2:100014. 10.1016/j.hfh.2022.100014.

[28] Kasirye F. The importance of needs in uses and gratification theory. Advance. 2022. 10.31124/advance.14681667.

[29] Sichach M. Uses and gratifications theory – background, history and limitations. SSRN Electron J. 2024. 10.2139/ssrn.4729248.

[30] Deci EL, Ryan RM. The what and why of goal pursuits: human needs and the self-determination of behavior. Psychol Inq. 2000;11(4):227–68. 10.1207/S15327965PLI1104_01.

[31] Cacioppo JT, Petty RE. The need for cognition. J Pers Soc Psychol. 1982;42(1):116–31. 10.1037/0022-3514.42.1.116.

[32] Seligman MEP, Flourish. New York: Simon & Schuster; 2011.

[33] Fredrickson BL. The role of positive emotions in positive psychology: the broaden-and-build theory of positive emotions. Am Psychol. 2001;56(3):218–26. 10.1037/0003-066X.56.3.218. 11315248 10.1037//0003-066x.56.3.218 PMC3122271

[34] Hobfoll SE. Conservation of resources: a new attempt at conceptualizing stress. Am Psychol. 1989;44(3):513–24. 10.1037/0003-066X.44.3.513. 2648906 10.1037//0003-066x.44.3.513

[35] Li L, Griffiths MD, Mei S, Niu Z. Fear of missing out and smartphone addiction mediates the relationship between positive and negative affect and sleep quality among Chinese university students. Front Psychiatry. 2020;11:877. 10.3389/fpsyt.2020.00877. 33192635 10.3389/fpsyt.2020.00877 PMC7481466

[36] Sharifian N, Zahodne LB. Daily associations between social media use and memory failures: the mediating role of negative affect. J Gen Psychol. 2021;148(1):67–83. 10.1080/00221309.2020.1743228. 32281502 10.1080/00221309.2020.1743228 PMC8074877

[37] Hamilton JL, Do QB, Choukas-Bradley S, Ladouceur CD, Silk JS. Where it hurts the most: peer interactions on social media and in person are differentially associated with emotional reactivity and sustained affect among adolescent girls. Res Child Adolesc Psychopathol. 2021;49(2):155–67. 10.1007/s10802-020-00725-5. 33294963 10.1007/s10802-020-00725-5 PMC7856166

[38] Lepp A, Barkley JE. The experimental effect of social media use, treadmill walking, studying, and a control condition on positive and negative affect in college students. Curr Psychol. 2023;42(30):26331–40. 10.1007/s12144-022-03747-y. 10.1007/s12144-022-03747-y PMC9483494 36157939

[39] Politte-Corn M, Dickey L, Abitante G, Pegg S, Bean CAL, Kujawa A. Social media use as a predictor of positive and negative affect: an ecological momentary assessment study of adolescents with and without clinical depression. Res Child Adolesc Psychopathol. 2024;52(5):743–55. 10.1007/s10802-024-01177-x. 38376716 10.1007/s10802-024-01177-x PMC11062812

[40] McRae K, Ochsner KN, Mauss IB, Gabrieli JJD, Gross JJ. Gender differences in emotion regulation: an fMRI study of cognitive reappraisal. Group Process Intergroup Relat. 2008;11(2):143–62. 10.1177/1368430207088035. 29743808 10.1177/1368430207088035 PMC5937254

[41] Whittle S, Simmons JG, Allen NB. Emotion and gender-specific neural processing in men and women. In: Legato MJ, editor. Principles of gender-specific medicine. Elsevier; 2017. pp. 183–201. 10.1016/B978-0-12-803506-1.00013-9.

[42] Lungu O, Potvin S, Tikàsz A, Mendrek A. Sex differences in effective fronto-limbic connectivity during negative emotion processing. Psychoneuroendocrinology. 2015;62:180–8. 10.1016/j.psyneuen.2015.08.012. 26318628 10.1016/j.psyneuen.2015.08.012

[43] Sharma R, Cameron A, Fang Z, Ismail N, Smith A. The regulatory roles of progesterone and estradiol on emotion processing in women. Cogn Affect Behav Neurosci. 2021;21(5):1026–38. 10.3758/s13415-021-00908-7. 33982247 10.3758/s13415-021-00908-7

[44] Chaplin TM. Gender and emotion expression: a developmental contextual perspective. Emot Rev. 2015;7(1):14–21. 10.1177/1754073914544408. 26089983 10.1177/1754073914544408 PMC4469291

[45] Guimond S, Chatard A, Lorenzi-Cioldi F. The social psychology of gender across cultures. The SAGE handbook of gender and psychology. SAGE Publications Ltd; 2013. pp. 216–33. 10.4135/9781446269930.n14.

[46] Schöne JP, Garcia D, Parkinson B, Goldenberg A. Negative expressions are shared more on Twitter for public figures than for ordinary users. PNAS Nexus. 2023;2(7):pgad219. 10.1093/pnasnexus/pgad219. 37457891 10.1093/pnasnexus/pgad219 PMC10338895

[47] Kelly MM, Tyrka AR, Price LH, Carpenter LL. Sex differences in the use of coping strategies: predictors of anxiety and depressive symptoms. Depress Anxiety. 2008;25(10):839–46. 10.1002/da.20341. 17603810 10.1002/da.20341 PMC4469465

[48] Matud MP. Gender differences in stress and coping styles. Pers Individ Dif. 2004;37(7):1401–15. 10.1016/j.paid.2004.01.010.

[49] Basińska MA, Kruczek A, Borzyszkowska A, Góralska K, Grzankowska I, Sołtys M. Flexibility in coping with stress questionnaire: structure and psychometric properties. Curr Issues Pers Psychol. 2021;9(2):179–94. 10.5114/cipp.2021.106412. 10.5114/cipp.2021.106412 PMC10658843 38013798

[50] Oliveira AJ, Lopes CS, Rostila M, Werneck GL, Griep RH, de Leon ACMP, et al. Gender differences in social support and leisure-time physical activity. Rev Saude Publica. 2014;48(4):602–12. 10.1590/S0034-8910.2014048005183. 25210819 10.1590/S0034-8910.2014048005183 PMC4181105

[51] Fischer AH, Rodriguez Mosquera PM, van Vianen AEM, Manstead ASR. Gender and culture differences in emotion. Emotion. 2004;4(1):87–94. 10.1037/1528-3542.4.1.87. 15053728 10.1037/1528-3542.4.1.87

[52] Sprecher S, Sedikides C. Gender differences in perceptions of emotionality: the case of close heterosexual relationships. Sex Roles. 1993;28(9–10):511–30. 10.1007/BF00289678.

[53] Ahmed M. Psychological impact of social media addiction on interpersonal relationships in Pakistan. Int J Psychol. 2023;8(4):53–65. 10.47604/ijp.2421.

[54] Satici B, Kayis AR, Griffiths MD. Exploring the association between social media addiction and relationship satisfaction: psychological distress as a mediator. Int J Ment Health Addict. 2023;21(4):2037–51. 10.1007/s11469-021-00658-0.

[55] Yang X, Liao T, Wang Y, Ren L, Zeng J. The association between digital addiction and interpersonal relationships: a systematic review and meta-analysis. Clin Psychol Rev. 2024;114:102501. 10.1016/j.cpr.2024.102501. 39265317 10.1016/j.cpr.2024.102501

[56] Hassan R, Mahmud S, Hasan K. Social media addiction and its consequences among youth: a developing country perspective. Glob Bus Rev. 2024. 10.1177/09721509241276720.

[57] Perez-Lozano D, Saucedo Espinosa F. Social media addiction: challenges and strategies to promote media literacy. In: Social media and modern society. IntechOpen; 2024. 10.5772/intechopen.1006166

[58] Kolhar M, Kazi RNA, Alameen A. Effect of social media use on learning, social interactions, and sleep duration among university students. Saudi J Biol Sci. 2021;28(4):2216–22. 10.1016/j.sjbs.2021.01.010. 33911938 10.1016/j.sjbs.2021.01.010 PMC8071811

[59] Nikolinakou A, Phua J, Kwon ES. What drives addiction on social media sites? The relationships between psychological well-being states, social media addiction, brand addiction and impulse buying on social media. Comput Hum Behav. 2024;153:108086. 10.1016/j.chb.2023.108086.

[60] Qiu R, Li Y, Gong Y, Guo Z, Cheng S, Li M, et al. Anxiety mediates the effect of social media addiction on negative attentional bias: the moderating role of impulsivity. Front Psychiatry. 2025;16:1592132. 10.3389/fpsyt.2025.1592132. 40656044 10.3389/fpsyt.2025.1592132 PMC12246940

[61] Varchetta M, Tagliaferri G, Mari E, Quaglieri A, Cricenti C, Giannini AM, et al. Exploring gender differences in internet addiction and psychological factors: a study in a Spanish sample. Brain Sci. 2024;14(10):1037. 10.3390/brainsci14101037. 39452049 10.3390/brainsci14101037 PMC11505988

[62] Amirthalingam J, Khera A. Understanding social media addiction: a deep dive. Cureus. 2024;16(10):e72499. 10.7759/cureus.72499. 39600781 10.7759/cureus.72499 PMC11594359

[63] Pellegrino A, Stasi A, Bhatiasevi V. Research trends in social media addiction and problematic social media use: a bibliometric analysis. Front Psychiatry. 2022;13:1017506. 10.3389/fpsyt.2022.1017506. 36458122 10.3389/fpsyt.2022.1017506 PMC9707397

[64] Valkenburg PM. Social media use and well-being: what we know and what we need to know. Curr Opin Psychol. 2022;45:101294. 10.1016/j.copsyc.2021.12.006. 35016087 10.1016/j.copsyc.2021.12.006

[65] Wu S. The influence of pessimism on adverse network behavior during COVID-19: the mediating effect of negative affect and risk perception. Curr Psychol. 2024;43(15):14027–36. 10.1007/s12144-022-03584-z. 10.1007/s12144-022-03584-z PMC9446659 36090911

[66] Md. Din NSB, Ahmad M. Emotional regulation on negative affect and aggression: a review. Asian People J. 2021;4(2):29–44. 10.37231/apj.2021.4.2.281.

[67] Kernkraut AM, Diniz Nagem Janot, de Matos L. The impact of negative affects on the high prevalence of anxiety, depression and stress in healthcare professionals. Work. 2024;79(2):857–866. 10.3233/WOR-230257 10.3233/WOR-230257 38701170

[68] Rutter LA, ten Thij M, Lorenzo-Luaces L, Valdez D, Bollen J. Negative affect variability differs between anxiety and depression on social media. PLoS ONE. 2024;19(2):e0272107. 10.1371/journal.pone.0272107. 38381769 10.1371/journal.pone.0272107 PMC10881019

[69] Burckhardt CS, Anderson KL. The quality of life scale (QOLS): reliability, validity, and utilization. Health Qual Life Outcomes. 2003;1(1):60. 10.1186/1477-7525-1-60. 14613562 10.1186/1477-7525-1-60 PMC269997

[70] Cotigă MI. Development and validation of a Romanian version of the expanded version of positive and negative affect schedule (PANAS-X). Procedia Soc Behav Sci. 2012;33:248–52. 10.1016/j.sbspro.2012.01.121.

[71] Watson D, Clark LA. The PANAS-X: manual for the positive and negative affect Schedule – Expanded form. University of Iowa; 1994. 10.17077/48vt-m4t2.

[72] Răscol M. The relation between quality of life, body image and anxiety among dietary supplements consumers. Rom J Cogn Behav Ther Hypn. 2017;4:3–4. https://www.rjcbth.ro/image/data/v4-i34/V4I3-4_Article_1_RJCBTH_2017.pdf.

[73] Hair JF, Black WC, Babin BJ, Anderson RE. Multivariate Data Analysis. 8th ed. Cengage; 2019.

[74] Kline RB. Principles and practice of structural equation modeling. 5th ed. Guilford Press; 2023.

[75] Al Nablsi E, Cordun M, Marinescu GA. The effect of inconsistent exercise on the functional parameters and body composition. Discobolul Phys Educ Sport Kinetother J. 2016;12(3):143–8.

[76] Albadi I, Ionescu EV, Iliescu MG, Onose G. Clinical study regarding outcomes of hydro-/ thermo-/ physio-kinesis therapy compared to those of physical-/ kinesis therapeutic procedures in spastic patients: preliminary results. Proc Rom Acad Ser B. 2020;22(3):147–64.

[77] Bistriceanu R, Mandu M, Carabageac D, Jianu C, Paraschiv R, Vlădulescu Trandafir AI, et al. Severe muscle skeletal trauma by car accident with upper brachial plexus injury favorable rehabilitative evolution. Rom J Med Rehabil Phys Med Balneoclimatol. 2024;1(2):131–5. 10.59277/RJMRPMB.2024.2.10.

[78] Bolboaşă IE. Aspecte psihologice privind relaţia dintre comportamentul suicidar și religiozitate. University of Bucharest, Faculty of Psychology and Educational Sciences; 2022.

[79] Chiriac GA. Metode și tehnici moderne de investigare a eficienței psihoterapiei integrative. University of Bucharest, Faculty of Psychology and Educational Sciences; 2019.

[80] Cheah JH, Amaro S, Roldán JL. Multigroup analysis of more than two groups in PLS-SEM: a review, illustration, and recommendations. J Bus Res. 2023;156:113539. 10.1016/j.jbusres.2022.113539.

[81] Dangbut A, Watcharamaisakul F, Champahom T, Jomnonkwao S, Wisutwattanasak P, Phojaem T, et al. The impact of attitude on high-speed rail technology acceptance among elderly passengers in urban and rural areas: a multigroup SEM analysis. Infrastructures. 2024;9(10):174. 10.3390/infrastructures9100174.

[82] Hu L, Bentler PM. Cutoff criteria for fit indexes in covariance structure analysis: conventional criteria versus new alternatives. Struct Equ Modeling. 1999;6(1):1–55. 10.1080/10705519909540118.

[83] Hair JF, Black WC, Babin BJ, Anderson RE. Multivariate data analysis. 8th ed. Boston: Cengage; 2019.

[84] Podsakoff PM, MacKenzie SB, Lee J-Y, Podsakoff NP. Common method biases in behavioral research: a critical review of the literature and recommended remedies. J Appl Psychol. 2003;88(5):879–903. 10.1037/0021-9010.88.5.879. 14516251 10.1037/0021-9010.88.5.879

[85] Podsakoff PM, MacKenzie SB, Podsakoff NP. Sources of method bias in social science research and recommendations on how to control it. Annu Rev Psychol. 2012;63:539–69. 10.1146/annurev-psych-120710-100452. 21838546 10.1146/annurev-psych-120710-100452

[86] Satorra A, Bentler PM. A scaled difference chi-square test statistic for moment structure analysis. Psychometrika. 2001;66(4):507–14. 10.1007/BF02296192. 10.1007/s11336-009-9135-y PMC2905175 20640194

[87] Yuan K-H, Bentler PM. Three likelihood-based methods for mean and covariance structure analysis with nonnormal missing data. Sociol Methodol. 2000;30(1):165–200. 10.1111/0081-1750.00078.

[88] Rosseel Y. Lavaan: an R package for structural equation modeling. J Stat Softw. 2012;48(2):1–36. 10.18637/jss.v048.i02.

[89] Shapiro SS, Wilk MB. An analysis of variance test for normality (complete samples). Biometrika. 1965;52(3–4):591–611. 10.1093/biomet/52.3-4.591.

[90] Spearman C. The proof and measurement of association between two things. Am J Psychol. 1904;172. 10.2307/1412159.

[91] Mardia KV. Measures of multivariate skewness and kurtosis with applications. Biometrika. 1970;57(3):519–30. 10.1093/biomet/57.3.519.

[92] Satorra A, Bentler PM. Corrections to test statistics and standard errors in covariance structure analysis. In: von Eye A, Clogg CC, editors. Latent variables analysis. Thousand Oaks: Sage; 1994. pp. 399–419.

[93] Breusch TS, Pagan AR. A simple test for heteroscedasticity and random coefficient variation. Econometrica. 1979;47(5):1287. 10.2307/1911963.

[94] Stebbins RA. Personal development through leisure. In: leisure’s legacy. Springer Int Publ. 2017;135–53. 10.1007/978-3-319-59794-2_10.

[95] Yi K, Luo H, Wei L. From the pitch to personal growth: investigating self-esteem as a mediator and parental support as a moderator in youth sports in China. Heliyon. 2024;10(10):e31047. 10.1016/j.heliyon.2024.e31047. 38770300 10.1016/j.heliyon.2024.e31047 PMC11103519

[96] Mertika A, Mitskidou P, Stalikas A. Positive relationships and their impact on wellbeing: a review of current literature. Psychol J Hellenic Psychol Soc. 2020;25(1):115. 10.12681/psy_hps.25340.

[97] Khanum F, Khan MA, Luqman MS. Role of recreational activities in the development of social attributes and learning skills of the students. Glob Phys Educ Sports Sci Rev. 2020;3(1):14–20. 10.31703/gpessr.2020(III-I).03.

[98] Nkwanyana S. Recreation and leisure in promoting social inclusion: a reflection of documented theory. Afr J Hosp Tour Leis. 2020;9(2):1-9 https://www.ajhtl.com/uploads/7/1/6/3/7163688/article_17_vol_9_2__2020_unizul.pdf

[99] Harman B, Kosirnik C, Antonini Philippe R. From social interactions to interpersonal relationships: influences on ultra-runners’ race experience. PLoS ONE. 2019;14(12):e0225195. 10.1371/journal.pone.0225195. 31790446 10.1371/journal.pone.0225195 PMC6886831

[100] Veenhoven R, Chiperi F, Kang X, Burger M. Happiness and consumption: a research synthesis using an online finding archive. Sage Open. 2021. 10.1177/2158244020986239.

[101] Helliwell JF, Putnam RD. The social context of well-being. Philos Trans R Soc Lond B Biol Sci. 2004;359(1449):1435–46. 10.1098/rstb.2004.1522. 15347534 10.1098/rstb.2004.1522 PMC1693420

[102] Hair JF, Ringle CM, Sarstedt M. PLS-SEM: indeed a silver bullet. J Mark Theory Pract. 2011;19(2):139–52. 10.2753/MTP1069-6679190202.

[103] Cheng C, Lau Y, Chan L, Luk JW. Prevalence of social media addiction across 32 nations: meta-analysis with subgroup analysis of classification schemes and cultural values. Addict Behav. 2021;117:106845. 10.1016/j.addbeh.2021.106845. 33550200 10.1016/j.addbeh.2021.106845

[104] Hou Y, Xiong D, Jiang T, Song L, Wang Q. Social media addiction: its impact, mediation, and intervention. Cyberpsychol J Psychosoc Res Cyberspace. 2019;13(1). 10.5817/CP2019-1-4.

[105] Sanzari CM, Gorrell S, Anderson LM, Reilly EE, Niemiec MA, Orloff NC, et al. The impact of social media use on body image and disordered eating behaviors: content matters more than duration of exposure. Eat Behav. 2023;49:101722. 10.1016/j.eatbeh.2023.101722. 37060807 10.1016/j.eatbeh.2023.101722 PMC10363994

[106] Twenge JM, Joiner TE, Rogers ML, Martin GN. Increases in depressive symptoms, suicide-related outcomes, and suicide rates among U.S. adolescents after 2010 and links to increased new media screen time. Clin Psychol Sci. 2018;6(1):3–17. 10.1177/2167702617723376.

[107] Twenge JM, Martin GN. Gender differences in associations between digital media use and psychological well-being: evidence from three large datasets. J Adolesc. 2020;79(1):91–102. 10.1016/j.adolescence.2019.12.018. 31926450 10.1016/j.adolescence.2019.12.018

[108] Çam E, Isbulan O. A new addiction for teacher candidates: social networks. Turk Online J Educ Technol. 2012;11:14–9.

[109] Ryan T, Chester A, Reece J, Xenos S. The uses and abuses of facebook: a review of Facebook addiction. J Behav Addict. 2014;3(3):133–48. 10.1556/JBA.3.2014.016. 25317337 10.1556/JBA.3.2014.016 PMC4189307

[110] Keles B, McCrae N, Grealish A. A systematic review: the influence of social media on depression, anxiety and psychological distress in adolescents. Int J Adolesc Youth. 2020;25(1):79–93. 10.1080/02673843.2019.1590851.

[111] Noguti V, Singh S, Waller DS. Gender differences in motivations to use social networking sites. In: social media marketing. IGI Global. 2018;680–95. 10.4018/978-1-5225-5637-4.ch034.

[112] Xu J. Motivations for social media use. Proceedings of the 2022 3rd international conference on mental health, education and human development (MHEHD 2022). Atlantis Press SARL; 2022. 10.2991/assehr.k.220704.071

[113] Longstreet P, Brooks S. Life satisfaction: a key to managing internet and social media addiction. Technol Soc. 2017;50:73–7. 10.1016/j.techsoc.2017.05.003.

[114] He L, Firdaus A, Gong J, Dharejo N, Aksar IA. How the social media impact women’s psychological well-being in the patriarchal structure? The moderating effect of social capital. BMC Public Health. 2024;24:581. 10.1186/s12889-024-18013-y. 38395820 10.1186/s12889-024-18013-y PMC10885406

[115] Krasnova H, Veltri NF, Eling N, Buxmann P. Why men and women continue to use social networking sites: the role of gender differences. J Strateg Inf Syst. 2017;26(4):261–84. 10.1016/j.jsis.2017.01.004.

[116] Azevedo MR, Araújo CLP, Reichert FF, Siqueira FV, da Silva MC, Hallal PC. Gender differences in leisure-time physical activity. Int J Public Health. 2007;52(1):8–15. 10.1007/s00038-006-5062-1. 17966815 10.1007/s00038-006-5062-1 PMC2778720

[117] Martinez-Gonzalez MA, Varo JJ, Santos JL, De Irala J, Gibney M, Kearney J, et al. Prevalence of physical activity during leisure time in the European Union. Med Sci Sports Exerc. 2001;33(7):1142–6. 11445761 10.1097/00005768-200107000-00011

[118] Monteiro CA, Conde WL, Matsudo SM, Matsudo VR, Bonseñor IM, Lotufo PA. A descriptive epidemiology of leisure-time physical activity in Brazil, 1996–1997. Rev Panam Salud Publica. 2003;14(4):246–54. 10.1590/S1020-49892003000900005. 14662075 10.1590/s1020-49892003000900005

[119] Fernandez-Lasa U, Eizagirre-Sagastibeltza O, Cayero R, Romaratezabala E, Martínez-Abajo J, Usabiaga O. Young women’s leisure time physical activity determinants: a mixed methods approach. Front Psychol. 2024;15:1281681. 10.3389/fpsyg.2024.1281681. 38434946 10.3389/fpsyg.2024.1281681 PMC10904608

[120] Tugade MM, Fredrickson BL. Resilient individuals use positive emotions to bounce back from negative emotional experiences. J Pers Soc Psychol. 2004;86(2):320–33. 10.1037/0022-3514.86.2.320. 14769087 10.1037/0022-3514.86.2.320 PMC3132556

[121] Mak AS, Mueller J. Negative affectivity, perceived occupational stress, and health during organisational restructuring: a follow-up study. Psychol Health. 2001;16(1):125–37. 10.1080/08870440108405494.

[122] Lyubomirsky S, King L, Diener E. The benefits of frequent positive affect: does happiness lead to success? Psychol Bull. 2005;131(6):803–55. 10.1037/0033-2909.131.6.803. 16351326 10.1037/0033-2909.131.6.803

[123] Drach RD, Orloff NC, Hormes JM. The emotion regulatory function of online social networking: preliminary experimental evidence. Addict Behav. 2021;112:106559. 10.1016/j.addbeh.2020.106559. 32768792 10.1016/j.addbeh.2020.106559

[124] Bayrami M, Abad THN, Ghoradel JA, Daneshfar S, Heshmati R, Moslemifar M. The role of positive and negative affectivity, optimism, pessimism, and information processing styles in student psychological adjustment. Procedia Soc Behav Sci. 2012;46:306–10. 10.1016/j.sbspro.2012.05.111.

[125] Lopez RB, Denny BT. Negative affect mediates the relationship between use of emotion regulation strategies and general health in college-aged students. Pers Individ Dif. 2019;151:109529. 10.1016/j.paid.2019.109529.

[126] Sultson H, Murd C, Havik M, Konstabel K. Negative affect instability predicts elevated depressive and generalized anxiety disorder symptoms even when negative affect intensity is controlled for: an ecological momentary assessment study. Front Psychol. 2024;15:1371115. 10.3389/fpsyg.2024.1371115. 38716268 10.3389/fpsyg.2024.1371115 PMC11074391

[127] Ahmad R, Hassan S, Ghazali NN, Al-Mashadani ARFS. The insta-comparison game: the relationship between social media use, social comparison, and depression. Procedia Comput Sci. 2024;234:1053–60. 10.1016/j.procs.2024.03.099.

[128] Qiu Y. Social comparison on social media platforms: a media and communication perspective. SHS Web Conf. 2024;185:03008. 10.1051/shsconf/202418503008.

[129] Eagly AH, Makhijani MG, Klonsky BG. Gender and the evaluation of leaders: a meta-analysis. Psychol Bull. 1992;111(1):3–22. 10.1037/0033-2909.111.1.3.

[130] Eagly AH, Nater C, Miller DI, Kaufmann M, Sczesny S. Gender stereotypes have changed: a cross-temporal meta-analysis of U.S. public opinion polls from 1946 to 2018. Am Psychol. 2020;75(3):301–15. 10.1037/amp0000494. 31318237 10.1037/amp0000494

[131] Woolley AW, Chabris CF, Pentland A, Hashmi N, Malone TW. Evidence for a collective intelligence factor in the performance of human groups. Science. 2010;330(6004):686–8. 10.1126/science.1193147. 20929725 10.1126/science.1193147

[132] O’Rourke T, Vogel C, John D, Pryss R, Schobel J, Haug F, et al. The impact of coping styles and gender on situational coping: an ecological momentary assessment study with the mHealth application trackyourstress. Front Psychol. 2022;13:913125. 10.3389/fpsyg.2022.913125. 35795429 10.3389/fpsyg.2022.913125 PMC9252427

[133] Proudfoot J, Fogarty AS, McTigue I, Nathan S, Whittle EL, Christensen H, et al. Positive strategies men regularly use to prevent and manage depression: a National survey of Australian men. BMC Public Health. 2015;15:1135. 10.1186/s12889-015-2478-7. 26573270 10.1186/s12889-015-2478-7 PMC4647287

[134] Gugushvili N, Täht K, Rozgonjuk D, Raudlam M, Ruiter R, Verduyn P. Two dimensions of problematic smartphone use mediate the relationship between fear of missing out and emotional well-being. Cyberpsychol J Psychosoc Res Cyberspace. 2020;14(2):article3. 10.5817/CP2020-2-3.

[135] Bartwal J, Nath B. Evaluation of nomophobia among medical students using smartphone in North India. Med J Armed Forces India. 2020;76(4):451–5. 10.1016/j.mjafi.2019.03.001. 33162655 10.1016/j.mjafi.2019.03.001 PMC7606098

[136] Rosenberg M. Society and the adolescent self-image. Princeton University Press; 1965.

[137] Gross JJ. The emerging field of emotion regulation: an integrative review. Rev Gen Psychol. 1998;2(3):271–99. 10.1037/1089-2680.2.3.271.

[138] Przybylski AK, Murayama K, DeHaan CR, Gladwell V. Motivational, emotional, and behavioral correlates of fear of missing out. Comput Hum Behav. 2013;29(4):1841–8. 10.1016/j.chb.2013.02.014.

